# Development and application of a SYBR green RT-PCR for first line screening and quantification of porcine sapovirus infection

**DOI:** 10.1186/1746-6148-8-193

**Published:** 2012-10-17

**Authors:** Axel Mauroy, Wim H M Van der Poel, Renate Hakze-Van der Honing, Christine Thys, Etienne Thiry

**Affiliations:** 1 Veterinary Virology and Animal Viral Diseases, Department of Infectious and Parasitic Diseases, Faculty of Veterinary Medicine, University of Liège, Liège, Belgium; 2 Department of Virology, Central Veterinary Institute, Wageningen University and Research Centre, Lelystad, the Netherlands

**Keywords:** Sapovirus, Diagnosis, Real time PCR, Porcine, SYBR green, Phylogeny

## Abstract

**Background:**

Sapoviruses are single stranded positive sense RNA viruses belonging to the family *Caliciviridae*. The virus is detected in different species including the human and the porcine species as an enteric pathogen causing asymptomatic to symptomatic enteritis. In this study, we report the development of a rapid real time qRT-PCR based on SYBR Green chemistry for the diagnosis of porcine sapovirus infection in swine.

**Results:**

The method allows the detection of porcine sapoviruses and the quantification of the genomic copies present in stool samples. During its development, the diagnostic tool showed good correlation compared with the gold standard conventional RT-PCR and was ten-fold more sensitive. When the method was applied to field samples, porcine noroviruses from genogroup 2 genotype 11b were also detected. The method was also applied to swine samples from the Netherlands that were positive for PoSaV infection. Phylogenetic results obtained from the samples showed that PoSaV sequences were genetically related to the currently described genogroup III, to the proposed genogroup VII and also to the MI-QW19 sequence (close to the human SaV sequences).

**Conclusions:**

A rapid, sensitive, and reliable diagnosis method was developed for porcine sapovirus diagnosis. It correlated with the gold standard conventional RT-PCR. Specificity was good apart for genogroup 2 genotype 11b porcine noroviruses. As a first line screening diagnosis method, it allows a quicker and easier decision on doubtful samples.

## Background

Sapoviruses (SaV) belong to the genus *Sapovirus* within the family *Caliciviridae*. These small (~30 nm), icosahedric, non-enveloped viruses have a single-stranded, positive sense, RNA genome. Two open reading frames (ORF) are classically described in the SaV genome wherein ORF1 codes the non-structural proteins and the single capsid protein and ORF2 codes a small and poorly known structural protein [1].

To date, no consensus method has been officially approved for rapid strain genogrouping within the genus *Sapovirus*. Genogrouping methods were initially predominantly based on molecular and phylogenetic analyses performed on sequences belonging to the viral RNA dependent RNA polymerase (RdRp) gene within ORF1, but such methods can now also be performed on complete capsid nucleotide sequences [2]. Currently, five genogroups (GG) are described in the genus *Sapovirus* (I-V) [3,4]. SaV have been identified in different animal species including human (Hu), porcine (Po), canine, bat and mink. Porcine sapoviruses (PoSaV) are mainly found in GGIII but two non-officially accepted GG (VI and VII) were proposed for classifying newly detected and sufficiently genetically divergent PoSaV sequences, and several other GG have been proposed [5-9]. Classical methods for the diagnosis of SaV infection are based on conventional or real-time RT-PCR, which target the conserved regions of the viral RdRp gene [10,11]. In this approach, the p289-290 pan-calicivirus primer pair [12] is often used as a first line diagnosis method to screen samples.

In humans, HuSaV are known as benign enteric pathogens, especially in paediatric and elderly gastroenteritis [13,14]. In swine, PoSaV cause mild or asymptomatic enteritis, mainly in piglets and post-weaning pigs [8,15,16]. Detection of PoSaV in swine samples is increasing in all parts of the world and their prevalence can be relatively high in swine premises [16]. PoSaV sequences have been geographically widely detected [17-21]. In Europe, PoSaV sequences were recently detected in about 50% of tested samples from six countries (Denmark, Spain, Slovenia, Italy, Finland and Hungary) with a molecular prevalence of 7.6% in all the samples [7]. Alongside this situation of rising levels of SaVs in the veterinary world lies the human context where, in Europe, SaV infections are also being increasingly reported [22]. Despite the fact that animal SaVs had previously never been detected in human beings or *vice versa*, several questions have been raised regarding the zoonotic risk or the potency of animal species as reservoir hosts or carriers of HuSaV [23]. The clinical and economic impacts of PoSaV on swine health and production are poorly understood and further study in this area is needed. Taken together, these data highlight the need for rapid, sensitive, specific and cost effective diagnostic tools to detect PoSaV infection.

Here, we report the development of a real time RT-PCR for the diagnosis and quantification of PoSaV infection in porcine stool. Based on broad range primers, SYBR Green chemistry and melting curve analysis, this diagnostic tool allows the quick discrimination between PoSaV, HuSaV and sequences from some other caliciviruses from the genera *Norovirus* (NoV) (except GII.11b PoNoV) and *Vesivirus*. Phylogenetic relationships of sequences detected in swine samples from the Netherlands with the new diagnostic tool are also presented.

## Methods

### Stool samples

Forty-three swine faecal samples were gathered as already described in a previous study [18]. Briefly, stools were sampled from young and adult pigs from different swine premises in Flanders (Belgium), where the animals showed clinical/necropsy signs of enteritis. The samples were gathered over a period of three months (June, July, August 2007). Other swine stool samples (n=111) originated from the Netherlands. A first series of 18 samples, tested by the Central Veterinary Institute (CVI, Lelystad, the Netherlands), had a previously known (but not communicated before testing) status for PoSaV and PoNoV infection, while the remaining 93, sampled from finishing pigs at slaughterhouse, had an unknown status. Positive controls for PoSaV strains belonging to GGVII and VIII were kindly provided by the CVI.

Bovine stool samples positive for bovine norovirus (BoNoV) and bovine kobuvirus (BoKoV) sequences had been gathered during previous studies [24-26]. Human stool samples positive for norovirus (HuNoV) or HuSaV were either gathered during studies in the laboratory (confirmed after RT-PCR amplification either by NoV-specific or pan-calicivirus primers and sequencing reactions), or were kindly provided by Dr P. Huynen (ULg) and the CVI (HuSaV GI.2). Feline calicivirus (FCV)-positive samples were sampled with nasal swabs on cats showing clinical signs of upper respiratory tract disease or in stool of dogs and cats with enteritis. These samples were confirmed as positive by RT-PCR with FCV-specific primers and sequencing reactions.

### RNA extractions and conventional RT-PCR

The QIAamp Viral RNA Mini kit (Qiagen GmbH, Hilden, Germany) was used to perform RNA extractions on the Belgian samples and samples with known status from the Netherlands, using ten grams of stool ten-fold diluted in PBS complemented with 0.01% sodium azide. RNA extractions on the remaining 93 samples from the Netherlands were performed after a ten-fold dilution in PBS using the High Pure RNA Isolation kit (Roche, Mannheim, Germany).

Two microlitres of each viral RNA extraction was subjected to a one-step RT-PCR reaction using the Quick Access kit (Promega, Madison, WI, USA) in a mix containing the p289-290 primers [12] (400 nM final concentration; p290: GATTACTCCAAGTGGGACTCCAC; p289: TGACAATGTAATCATCACCATA) and bovine serum albumin (BSA, 400 ng/μl final concentration). Reverse transcription was performed at 48°C for 10 min and the PCR parameters were as follows: 3 min at 95°C, 45 cycles of 1 min at 95°C, 45 sec at 51°C and 45 sec at 68°C, and 7 min at 68°C for the final extension step.

RT-PCR amplicons were purified from agarose gel with the QIAquick purification kit (Qiagen GmbH, Hilden, Germany) or by precipitation, and were directly sequenced twice or cloned (PC45, 46, 47, 48, 49 and 63) into pGEM-Teasy (Promega, Madison, WI, USA) before sequencing. Plasmid DNA was purified on three clones for each previously mentioned amplicon with the Miniprep kit (Invitrogen, Carlsbad, CA, USA). Sequencing reactions were carried out with BigDye terminator kit version 3.1 and resolved with an ABI 3730 automatic capillary sequencer (AppliedBiosystem, Foster City, CA, USA).

### Bioinformatics

Sequences were analysed with the BioEdit Sequence Editor version 7.0 software [27]. Nucleotidic similarity with the NCBI genetic database was assessed using the BLAST tool (available at http://www.ncbi.nlm.nih.gov/blast/Blast.cgi). Phylogenetic inference was performed with the MEGA version 4 software package [28]. Phylogenetic trees were constructed by neighbor-joining analysis where evolutionary distances were computed using the Maximum Composite Likelihood method. The confidence values of the internal nodes were calculated by performing bootstrap analyses with 1,000 replicates.

### Standard of quantification

A p289-290 amplicon, amplified on a canine sample, was cloned into pGEMt-Easy (pSTD) (Promega, Madison, WI, USA) and sequenced at the GIGA facilities of the University of Liège. Sequencing was carried out with the BigDye terminator kit version 3.1 and resolved with an ABI 3730 automatic capillary sequencer (AppliedBiosystem, Foster City, CA, USA). The p289-290 amplicon was then *in vitro* transcribed with the Ribomax kit (Promega, Madison, WI, USA) following the manufacturer’s instructions. Briefly, 4 μg of pSTD were linearised with the *SpeI* restriction endonuclease and purified with phenol:chloroform:isoamyl alcohol (25:24:1). DNA was precipitated with 3M Na acetate and ethanol purified, and the concentration was then measured on a Nanodrop 1000 spectrophotometer (Technilab). The transcription was performed on 10 μg of DNA with T7 RNA polymerase and the reaction mix was then treated with 5U DNAse for 1 h at 37°C. The transcribed RNA was controlled for quality and purity (DNA absence) by both RT-PCR (Quick Access kit, Promega, Madison, WI, USA) and PCR (Taq polymerase from Westburg, Leusden, the Netherlands) with the p289-290 primers. RNA concentration was measured on a Nanodrop 1000 spectrophotometer (Technilab). Genomic copies were deduced and serial ten-fold dilutions were prepared with ultrapure RNAse free H_2_O (Invitrogen, Carlsbad, USA). Aliquots of the master stock were stored at −80°C and measured once again before dilution and use.

### SYBR green qRT-PCR

The following mixture was constituted using the iScript One-Step RT-PCR kit for SYBR Green assay (Biorad, Nazareth, Belgium): p289-290 primers (300 nM each), 0.5 μl enzyme mix, 12.5 μl reaction buffer, BSA (400 ng/μl final concentration), 2 μl of RNA extraction, ultrapure RNAse free H_2_O (Invitrogen, Carlsbad, USA) to 25 μl. The protocol included a reverse transcription step of 18 min at 48°C, an initial denaturation step of 5 min at 95°C, 45 cycles of 10 sec at 95°C, 20 sec at 51°C, 45 sec at 60°C and a final extension step of 3 min at 95°C. Data were obtained during the elongation period. Melting curve analysis was performed after RT-PCR reaction at a start- and an end-temperature of 65°C and 95°C respectively. The real time RT-PCR reactions were carried out on an iCycler thermocycler (Biorad, Nazareth, Belgium).

### Evaluation of the assay parameters

#### Assay development

RNA extractions from three PoSaV-positive samples (named PC34, PC29 and PC42) were selected on the basis of phylogenetic analysis performed on partial RdRp sequences during a previous study [18], RNA extractions from three PoSaV-positive samples were selected: PC34, PC29 and PC42. Their sequences were genetically related to GGIII, and to the proposed GGVI and proposed GGVII respectively. RNA extractions from two PoNoV-positive swine samples (PC23, PC26) [18], two HuSaV- (Be1, Be2) and two HuNoV-positive human samples (ISP475, CrH2) were also incorporated into the melting curve study.

#### Assay repeatability and reproducibility

Assay repeatability and reproducibility were established by determination of the mean melting temperature, standard deviation and coefficient of variation on the previously described 3 PoSaV-, 2 HuSaV-, 2 HuNoV- and 2 PoNoV-positive samples, in three different assays, three times in each assay.

#### Assay specificity

The specificity of the test was determined by comparison of the melting temperature obtained on 7 HuNoV-, 6 feline calicivirus-, 6 BoNoV-, 1 HuSaV-, 6 PoNoV- and 3 BoKoV-positive samples with those obtained on GGIII, GGVII and GGVIII PoSaV-positive samples.

#### Relative sensitivity and specificity of the assay

Relative sensitivity and relative specificity of the SYBR Green real-time RT-PCR were tested first in a blind test, on 18 samples from Dutch swines (PC44 to PC63) where 6 had been previously diagnosed as PoSaV-positive, 7 PoNoV-positive and the five remaining samples as negative for both viruses.

#### Genomic quantification and detection limit

Genomic quantification was carried out by running samples of the standard curve with unknown samples. Positive samples from the previously tested 18 Dutch samples were also quantified. The detection limit was established by both conventional and real-time analysis on a serial ten-fold dilution of sample PC29 in RNAse free water. The same assay was also performed on serial dilutions of PC29 in PC16 (PoSaV-negative sample, [18]).

### Assay application to field samples

The newly developed test was subsequently performed on 93 Dutch porcine samples with an unknown status for calicivirus infection. These samples were also tested in parallel using conventional RT-PCR (the gold standard) with the p289-290 primers.

## Results

### Assay development

The mean melting temperatures, recorded by the Biorad thermocycler throughout the nine assays for the three different PoSaV were 87.28°C ± 0.25 (PC34), 86.83°C ± 0.33 (PC29) and 85.39°C ± 0.33 (PC42). The melting temperature recorded in Be1 (HuSaV, 84.78°C ± 0.26) was close to that of PC42 (GGVII-related PoSaV), but it was quite different from the melting temperatures recorded in the other genetically related PoSaV. Melting temperatures registered on the second HuSaV (Be2), and on the HuNoV (ISP475, CrH2) and the PoNoV (PC23, PC26) were significantly different: 84.33°C ± 0.25, 82.33°C ± 0.25, 74.67°C ± 0.50, 77.89°C.

### Assay repeatability and reproducibility

The intra-assay and inter-assay variations in the melting curve temperature analysis were found to be very low, as shown by the calculated coefficients of variation (Table 1). Quantification of the genomic copies present in the samples was less reliable ( Additional file 1: Table S1).

**Table 1:** Determination of the melting temperature obtained on different samples (porcine sapovirus- or norovirus-positive swine samples, human sapovirus- or norovirus-positive human samples)

**Sample identifier**	**Infecting virus**	**GG**	**Intra-assay**									**Inter-assay**		
			**Assay 1**			**Assay 2**			**Assay 3**					
			**mean**	**SD**	**CV (%)**	**mean**	**SD**	**CV (%)**	**mean**	**SD**	**CV (%)**	**mean**	**SD**	**CV (%)**
PC34	PoSaV	III	87	0	0	87.5	0	0	87.33	0.29	0.33	87.28	0.26	0.30
PC29		VI	86.5	0	0	87	0	0	87	0	0	86.83	0.25	0.29
PC42		VII	85	0	0	85.5	0	0	85.67	0.29	0.34	85.39	0.33	0.39
PC23	PoNoV	II	77.5	0	0	78	0	0	78.17	0.29	0.37	77.89	0.33	0.43
PC26		II	79	1.73	2.19	81.5	0	0	78	0	0	79.5	1.79	2.25
Be1	HuSaV	I	84.5	0	0	85	0	0	84.83	0.29	0.34	84.78	0.26	0.31
Be2		I	84	0	0	84.5	0	0	84.5	0	0	84.33	0.25	0.30
ISP475	HuNoV	II	82	0	0	82.5	0	0	82.5	0	0	82.33	0.25	0.30
CrH2		II	74.33	0.76	1.03	75	0	0	74.67	0.29	0.39	74.67	0.5	0.67

Genogrouping is based on phylogenetic relationships in the polymerase sequence. GG: genogroup; PoSaV: porcine sapovirus; PoNoV: porcine norovirus; HuSaV: human sapovirus; HuNoV: human norovirus; SD: standard deviation; CV: coefficient of variation (%).

Intra- and inter-assay variability recorded for the SYBR green real-time RT-PCR.

### Assay specificity

Test specificity was established by testing on a panel of positive samples for other classical enteric caliciviruses that could be detected either in porcine species or in other species (human, feline, bovine). The obtained results showed significantly different melting temperatures between these samples and the PoSaV-positive samples from GGIII (PC33), VII and VIII (Table 2). Interestingly, melting temperatures from sequences non-genetically related to PoSaV were also significantly different from those previously obtained for PC34, PC29 and PC42. On the other hand, melting temperatures obtained in the GGVII and GGVIII genetically related sequences were found to be in the same range as those from PC34, PC29 and PC42.

**Table 2:** Specificity of the SYBR Green qRT-PCR based on melting curve temperature analysis

**Infecting virus**	Genogroup and genotype*	**Identifier**	**Melting temperature**
PoSaV	III	PC33	87.0
PoNoV	II	PC54	83.5
		PC55	83.5
		PC58	83.5
		PC59	83.5
		PC60	83.5
		PC61	83.5
HuNoV	I.4	Hu360	83.5
	II	HuJ	83.5
	II.2	Hu333	83.5
	II.4	Hu384	83.5
	II.4	Hu498	83.5
BoNoV	III.1	BV164	83.5
	Recombinant III.1/III.2	BV416	84.0
	III.2	BV52	83.5
	III.2	BV15	83.5
	III.2	BV253	83.5
	III.2	BV344	83.0
FCV	ND	F106	84.0
		F117	83.5
		F119	84.0
		F226	84.0
		F242	83.5
		F295	83.0

* Genogrouping and genotyping are based on phylogenetic relationships in polymerase sequence. PoSaV: porcine sapovirus; HuSaV: human sapovirus; PoNoV: porcine norovirus; HuNoV: human norovirus; BoNoV: bovine norovirus; FCV: feline calicivirus; BoKoV: bovine kobuvirus; ND: not done.

### Assay relative sensitivity/specificity

**Figure 1:**
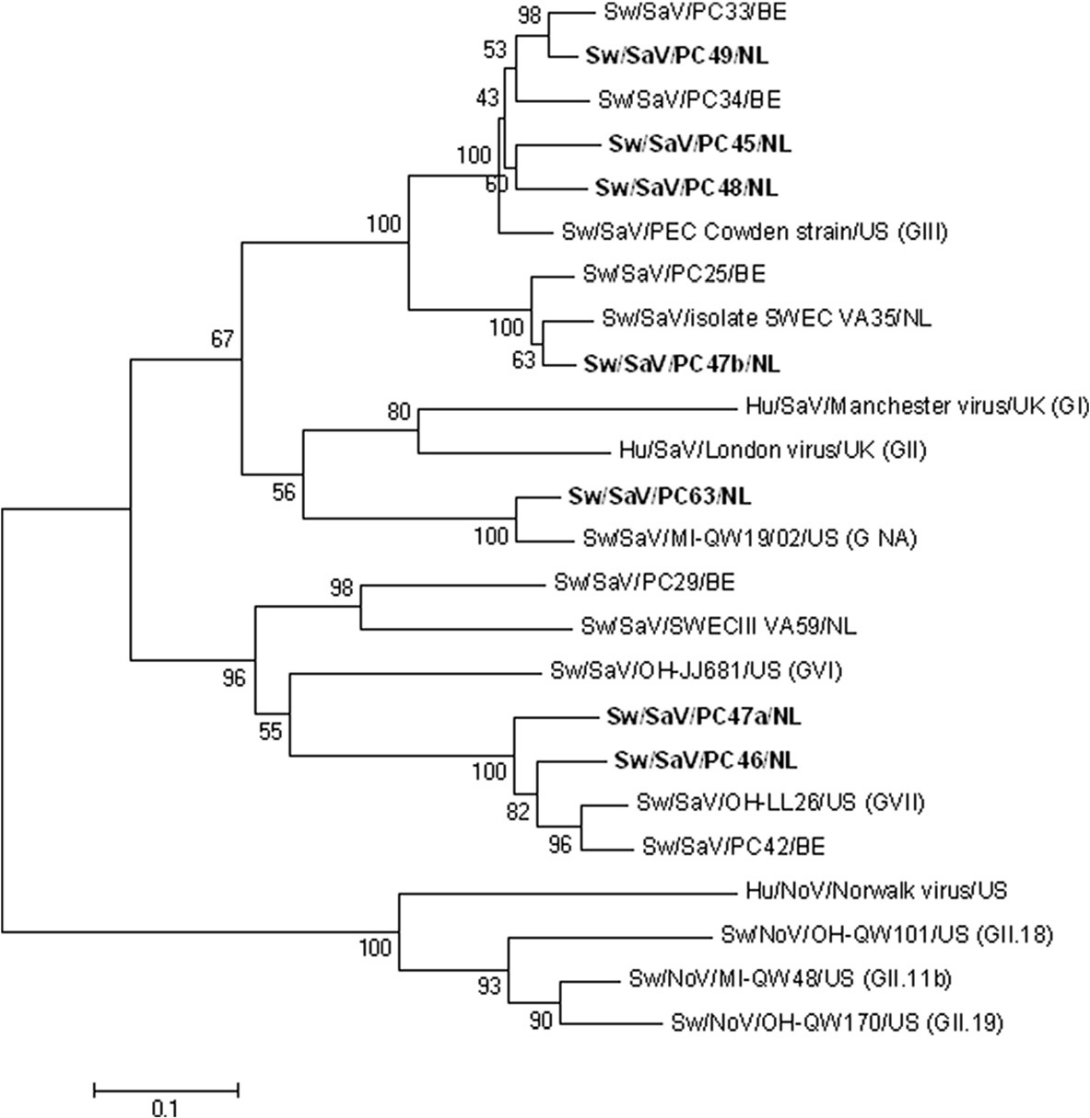
**Neighbor-joining phylogenetic tree based on partial RNA dependent RNA polymerase coding region (267 nt) of porcine sapovirus strains identified in this study (in bold face), human and porcine sapovirus reference strains, the Norwalk virus (human norovirus) and some published porcine norovirus strains.** Scale bar represents the phylogenetic distances expressed as units of expected nucleotide substitutions per site. Bootstrap values (1,000 replicates) are reported. Accession numbers in GenBank: Sw/NoV/OH-QW170/US [AY823306], Sw/NoV/MI-QW48/US [AY823303], Sw/NoV/OH-QW101/US [AY823304], Hu/NoV/Norwalk/US [M87661], Sw/SaV/SWECIII VA59/NL [AY615813], Hu/SaV/Manchester/UK [X86560], Hu/SaV/London/UK [U95645], Sw/SaV/MI-QW19/US [AY826424], Sw/SaV/SWEC VA35/NL [AY615808], Sw/SaV/PEC Cowden strain/US [AF182760], Sw/SaV/PC25/BE [EU652844], Sw/SaV/PC29/BE [EU652845], Sw/SaV/PC33/BE [EU652846], Saw/SaV/PC34/BE [EU652847], Sw/SaV/PC42 [EU652848], Sw/SaV/PC45/NL [JN644270], Sw/SaV/PC46/NL [JN644271], Sw/SaV/PC47a/NL [JN644275], Sw/SaV/PC47b/NL [JN644276], Sw/SaV/PC48/NL [JN644272], Sw/SaV/PC49/NL [JN644273], Sw/SaV/PC63/NL [JN644274]. NA: not assigned.

A test was then performed on extractions from the 18 Dutch samples with a previously tested, but not communicated, status. A very good correlation was found between the results obtained in the two different laboratories. Only two differences were found: sample PC44, tested as low positive in the first laboratory, was found to be negative either by conventional or real-time RT-PCR in the second laboratory and inversely for sample PC63 (Table 3). The amplicons detected in the PoSaV-positive samples were sequenced and were found to be genetically related to both GGIII and currently unclassified PoSaV (Figure 1). The observed melting temperatures were correlated with previous results found during the development of the assay. The highest melting temperatures were found for samples infected with GGIII PoSaV, while lower melting temperatures were found for PoSaV genetically related to currently unclassified PoSaV (proposed GGVI and VII).

**Table 3:** Test validation performed on viral RNA extractions with predetermined but unknown status

**Sample status**^**a**^	**Identifier**	**Test**				
		**Melting temperature**	**SYBR Green real time RT-PCR**	**Conventional RT-PCR**	**Sequencing**	**Quantification (genomic copies/μl of RNA extraction)**
PoSaV +	PC44^b^	80.5	-	-	ND	
	PC45	86.0	+	+	+	1.41E+05
	PC46	86.0	+	+	+	3.25E+04
	PC47	85.0	+	+	+	2.91E+04
	PC48	87.5	+	+	+	2.76E+05
	PC49	86.0	+	+	+	1.04E+06
PoNoV +	PC54	84.5	-	+/−^c^	-	
	PC55	80.0	-	+/−^c^	-	
	PC58	80.0	-	+/−^c^	-	
	PC59 ^b^	81.5	-	-	ND	
	PC60	80.5	-	+/−^c^	-	
	PC61	80.5	-	+/−^c^	-	
	PC62	84.5	-	+/−^c^	-	
Negative	PC52	81.5	-	-	ND	
	PC53	79.5	-	-	ND	
	PC56	80.0	-	-	ND	
	PC57	82.0	-	-	ND	
	PC63	85.0	+ ^d^	+	+	

^a^: Sample status was determined in another laboratory. Positive porcine sapovirus (PoSaV) samples were determined by conventional RT-PCR; positive porcine norovirus (PoNoV) samples were determined by real time RT-PCR.

^b^: Low positive in the reference laboratory.

^c^: Doubtful samples, as difference between molecular weights of amplicons obtained with p289/290 primer pair on sapovirus- and norovirus-positive amplicons were very small.

^d^: The sequencing reaction performed on that sample gave a sequence genetically related to the QW19-like, SWECII/VA103 and SWECII/VA14 porcine sapovirus strains. QW19 strains are known to share high amino acid identities with human sapovirus strains (Wang et al., 2005) and thus, QW19-related strains could provide slightly lower melting temperatures, such as those registered for human sapovirus.

Ct: Cycle threshold; ND: not done; PoSaV: porcine sapovirus; PoNoV: porcine norovirus.

### Genomic copy quantification and detection limit

**Figure 2:**
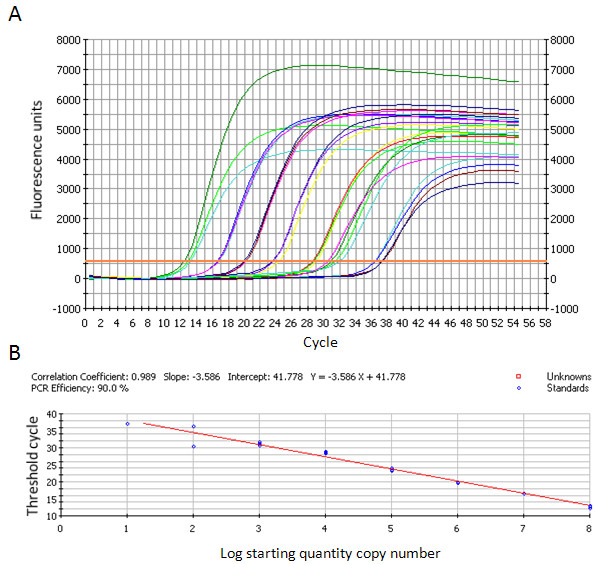
**Detection of ten-fold serial dilution of RNA transcripts by the SYBR Green real-time RT-PCR.A**: real time PCR curves. **B**: Standard curve where each dot represents the cycle threshold value recorded for each quantity of molecule (logarithmic representation).

**Figure 3:**
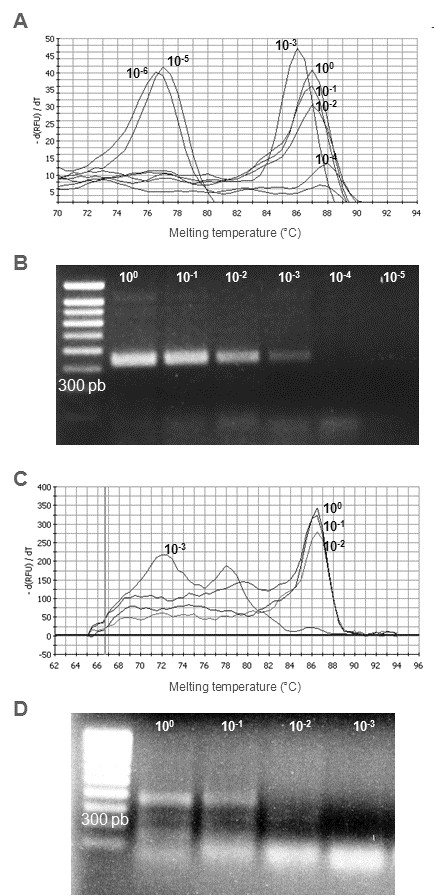
**Detection limit of the SYBR Green real-time RT-PCR (A, C) in comparison with those of conventional RT-PCR (B, D).** The two assays were performed on ten-fold dilutions of PC29 (from 10^0^ to 10^-6^ in RNAse free water or from 10^0^ to 10^-4^ in faecal suspension from a PoSaV-negative sample).

A very good correlation (R^2^=0.997) between the different dilutions of the standard curve was found for up to 10^3^ copies. The correlation was then found to be slightly lower (R^2^=0.989). However, quantification was attainable for up to 10 genomic copies (Figure 2), enabling us to quantify the genomic charge within the positive Dutch samples from the first series (known status) (Table 3). The real-time method was shown to be 10 times more sensitive than conventional RT-PCR on a serial ten-fold dilution of PC29 in RNAse free water (Figure 3A and B) or in faecal suspension from a PoSaV-negative sample (Figure 3C and D).

### Test application

**Figure 4:**
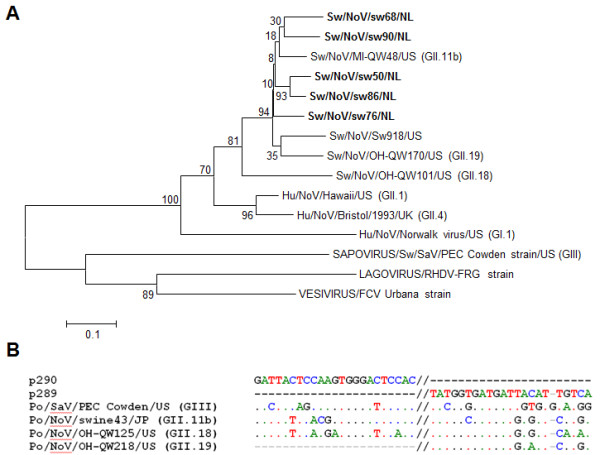
**A. Neighbor-joining phylogenetic tree based on partial RNA dependent RNA polymerase coding region (230 nt) of porcine norovirus strains identified in this study (in bold face), human and porcine norovirus reference strains, and other reference strains from the *Caliciviridae *family.** Scale bar represents the phylogenetic distances expressed as units of expected nucleotide substitutions per site. Bootstrap values (1,000 replicates) are reported. Accession numbers in GenBank: Sw/NoV/OH-QW170/US [AY823306], Sw/NoV/MI-QW48/US [AY823303], Sw/NoV/OH-QW101/US [AY823304], Sw/NoV/Sw918/JP [AB074893], Sw/SaV/PEC Cowden strain/US [AF182760], Hu/NoV/Norwalk/US [M87661], Hu/NoV/Hawaii/US [U07611], Hu/NoV/Bristol/UK [X76716], LAGOVIRUS/RHDV-FRG strain [M67473], VESIVIRUS/FCV Urbana strain [L40021], Sw/NoV/sw50/NL [JN644277], Sw/NoV/sw68/NL [JN644278], Sw/NoV/sw76/NL [JN644279], Sw/NoV/sw86/NL [JN644280], Sw/NoV/sw90/NL [JN644281]. **B**. Multiple alignment performed with ClustalW on different reference porcine and human strains from the *Sapovirus* and *Norovirus* genera. Results were compared with those of the p289-290 primer pair.

When the test was applied to 93 field samples with unknown status, 5 potentially positive results were found in real time SYBR green RT-PCR on the basis of their melting curve analysis. Conventional RT-PCR yielded similar results but with several other samples also giving amplicons near the expected molecular weight. Thus, diagnosis of calicivirus infection in conventional RT-PCR was clearly difficult based on the sole differentiation between these unexpected amplicons and the right ones. Following sequencing, the 5 positive amplicons were found to be genetically related to GII.11b PoNoV (Figure 4A). When reference porcine and human strains from both the *Sapovirus* and *Norovirus* genera were aligned and compared to the p289-290 primer pair, it was shown that primer hybridisation was higher for PoNoV from the GII.11b genotype than for strains from GII.18 (the same analysis was more difficult to perform on GII.19 strains as sequences covering the p290 region are not currently available in GenBank, Figure 4B).

## Discussion

Usage of the p289-290 primer pair allows a broad range diagnosis of calicivirus infections. However, this lack of specificity means amplicons obtained within conventional PCR require sequencing confirmation. In this study, a real time qRT-PCR, based on the p289-290 primer pair and SYBR green technology was developed, allowing the detection of SaV identifiable in stools from young and finishing pigs. The diagnostic tool showed a similar specificity but a higher sensitivity than conventional RT-PCR. During its development, the diagnostic tool presented here showed a good sensitivity against PoSaV from the GG currently described, and a good specificity against other viruses from the family *Caliciviridae* (HuSaV, HuNoV, PoNoV and FCV) and against those from genetically related families (kobuviruses). Reproducibility was validated intra- and interassay. The melting curve analysis that forms part of the diagnostic tool allows the discrimination between PoSaV genetically related to different genotypes (GGIII and the proposed GGVI, VII and VIII). Moreover, the assay allows the quantification of genomic copies present in the sample. The detection limit with the new diagnostic tool compared to conventional RT-PCR was determined as being ten-fold more sensitive. However, when applied to field samples, a subtle lack of specificity was found, as the tool was also able to detect another swine pathogen from a specific genotype: GGII.11b PoNoVs. Phylogenetic results are also presented for PoSaV strains detected in samples from the Netherlands.

In the first validation test (blind analysis of swine samples, with known but not communicated status), a good correlation was found between the results obtained with the test and the results obtained in another laboratory by conventional RT-PCR (diagnosis of PoSaV) and real time RT-PCR (diagnosis of PoNoV). Two differences were found: sample PC44 was weakly positive for PoSaV in the other laboratory and negative in both conventional and real time RT-PCR in our conditions, and the inverse was true for sample PC63. A loss of virus during storage or a failure in its genomic extraction could explain the first result. For the second case, inhibition during RT-PCR reaction or difference in the melting temperature used by the two laboratories could explain the result. For this last sample, the melting curve was also significantly different from those usually expected for PoSaV. This could be explained by the fact that PC63 is genetically related to the QW19-like, SWECII/VA103 and SWECII/VA14 PoSaV strains, known to share high nucleotidic identities with HuSaV [10]. That study was noteworthy in showing the circulation, in the Netherlands, of PoSaV from the previously described GGIII and VII but also those genetically related to sequences close to human strains (PC63). Remarkably, sequence analysis revealed co-infection in one sample (PC47), with significantly different sequences from each other and from those detected in other samples. To our knowledge, this is the first report of co-infection by PoSaV and confirms the well documented hypothesis regarding the recombination potential of these viruses [29,30].

In the second validation test (swine samples with an unknown status), 5 samples were found to be positive through melting curve analysis. Following sequencing reaction, no PoSaV sequences were detected in these samples. This could be explained by the fact that the samples came from swines at the slaughter house and that the highest prevalence of PoSaV infection is usually found in piglets aged 2 to 8 weeks [7]. These positive samples were found to contain PoNoV sequences and these sequences were all genetically related to the GGII.11b genotype. It was not surprising as the p289-290 primer pair was originally developed to react with both NoVs and PoSaVs [12]. But this represented a discordant result compared with those obtained during the development of the specificity test. This could be explained by the fact that the PoNoV-positive samples (PC23 and PC26) used were not genetically related to GGII.11b [18]. Also, in the first validation test, not all PoNoV-positive samples were detected. Unfortunately, the genotype of the PoNoV-positive samples provided was not clarified. Moreover, sequencing reaction performed on doubtful samples by conventional RT-PCR in our laboratory (PC54, 55, 58, 60, 61 and 62) gave no calicivirus-related sequences except for one amplicon in PC54, which was genetically related to GII.19 PoNoV. Bioinformatic analysis confirmed the easier annealing of the p289-290 pair on GII.11b-related sequences than on GII.18 PoNoV. This lack of specificity could be bypassed on the basis of the age of the animal at the time of sampling, as PoNoV infections have usually been found in older animals [31] or by usage in serie of a pan-calicivirus primer pair for confirmation, as the SR80(+)/JV33 primer pair [32] . This would enlarge the detection spectrum of the test but would strengthen the need for sequence determination in each PCR result. Overall, the present study shows that when the gold standard conventional RT-PCR was applied to the field samples, discrimination between amplicons found near the expected molecular weight and the right amplicons was sometimes difficult in comparison with the easier analysis allowed by melting curve analysis as part of the SYBR green qRT-PCR.

## Conclusions

The routine molecular diagnosis of calicivirus infection is based on the use of several different primer pairs such as p289-290. But the relative lack of specificity obtained through using this primer pair requires confirmation, such as the sequencing of the obtained amplicon. PoSaV diagnosis, currently based on conventional RT-PCR, requires a powerful, rapid, sensitive, specific and reliable tool, in order to investigate the epidemiological situation in Europe. These requirements are met in the SYBR green-based qRT-PCR presented here. To our knowledge, this is the first qRT-PCR assay developed for PoSaV diagnosis. When applied to field samples, a good correlation was found between this assay and the standard conventional RT-PCR. However, the newly developed test allows a quicker and easier decision on doubtful samples. A lack of specificity was shown regarding GGII.11b PoNoV but this allowed the detection of another important swine enteric pathogen in the same analysis. Interestingly, the obtained results and those from the literature raise a question regarding the apparently well-defined age spectrum of swine susceptibility to PoSaV and to PoNoV.

## Competing interests

None of the authors has any financial or personal relationships that could inappropriately influence or bias the content of the paper.

## Authors’ contributions

Conceived the study: AM. Performed the analysis: AM, RHvdH and CT. Analysed the data: AM, RHvdH and WvdP. Wrote the manuscript: AM. Reviewed the manuscript: WvdP and ET. All the authors read and approved the final manuscript.

## Supplementary Material

Additional file 1 **Table S1. **Determination of genomic copies on different porcine sapovirus- positive swine samples. Intra- and inter-assay variabilities were recorded in SYBR green real-time RT-PCR. Three measures were realised per assay. Means and standard deviations are expressed as genomic copies. (PDF 4 kb) Click here for file
