# Supplementary material for: Development and application of a SYBR green RT-PCR for first line screening and quantification of porcine sapovirus infection
Source: BMC Vet Res. 2012 Oct 17;8:193. doi: 10.1186/1746-6148-8-193 (PMC3528410; doi:10.1186/1746-6148-8-193)
Supplement: Additional file 1 — Table S1. Determination of genomic copies on different porcine sapovirus- positive swine samples. Intra- and inter-assay variabilities were recorded in SYBR green real-time RT-PCR. Three measures were realised per assay. Means and standard deviations are expressed as genomic copies. (PDF 4 kb) [file 1746-6148-8-193-S1.pdf]

Supplementary table I: Determination of genomic copies on different porcine sapovirus- positive swine samples. Intra- and inter-assay variabilities were recorded in SYBR green real-time RT-PCR. Three measures were realised per assay. Means and standard deviations are expressed as genomic copies.

Sample identifier	Infecting virus	GG	Intra-assay									Inter-assay		
			Assay 1			Assay 2			Assay 3					
			Mean	SD	CV(%)	mean	SD	CV(%)	mean	SD	CV(%)	mean	SD	CV(%)
PC34	PoSaV	III	2.31^{E}05	6.77^{E}04	29.3	6.16^{E}05	8.91^{E}04	14.5	1.43^{E}06	5.55^{E}05	38.8	7.59^{E}05	2.10^{E}05	40.3
PC29		VI	3.59^{E}05	1.09^{E}05	30.2	7.59^{E}05	1.01^{E}04	13.3	4.47^{E}05	1.36^{E}05	30.4	5.22^{E}05	6.12^{E}05	80.6
PC42		VII	3.14^{E}04	1.90^{E}04	60.5	2.52^{E}04	1.12^{E}04	44.6	4.42^{E}04	2.46^{E}04	55.7	3.36^{E}04	9.68^{E}03	28.8

Genogrouping is based on phylogenetic relationships in the polymerase sequence. GG : genogroup ; PoSaV : porcine sapovirus; SD : standard deviation ; CV : coefficient of variation.
